# Incidence and outcomes of FGFR inhibitor-associated retinopathy of patients treated with oral erdafitinib across the clinical trial program

**DOI:** 10.1093/oncolo/oyag174

**Published:** 2026-05-13

**Authors:** Anne O’Hagan, Arlene Siefker-Radtke, Yohann Loriot, Kris Deprince, Lauren Crow, Michal Laron, Ron Adelman, Hussein Sweiti, Spyros Triantos

**Affiliations:** Johnson & Johnson, Spring House, PA, 19477, United States; The University of Texas MD Anderson Cancer Center, Houston, TX, 77030, United States; Institut Gustave Roussy, Villejuif, 94805, France; Johnson & Johnson, Beerse, 2340, Belgium; Johnson & Johnson, Spring House, PA, 19477, United States; Johnson & Johnson, Milpitas, CA, 95054, United States; Mayo Clinic, Jacksonville, FL, 32224, United States; Johnson & Johnson, Spring House, PA, 19477, United States; Johnson & Johnson, Spring House, PA, 19477, United States

**Keywords:** fibroblast growth factor receptors, erdafitinib, retinopathy

## Abstract

**Background:**

Fibroblast growth factor receptors inhibitors (FGFRi) are approved treatments for various malignancies. FGFRi-associated retinopathy is a class effect of FGFRi, including erdafitinib, pemigatinib, infigratinib, and rogaratinib.

**Patients and Methods:**

We summarized FGFRi-associated retinopathy with erdafitinib across 5 clinical studies in patients with metastatic urothelial cancer (mUC pooled studies) and one study in patients with advanced solid tumors (RAGNAR). We also present changes in visual acuity, retinal pigment epithelial elevation, and subretinal fluid in RAGNAR.

**Results:**

One hundred and three (21.5%) of 479 patients in the mUC pooled studies and 43/314 (13.7%) patients in RAGNAR experienced FGFRi-associated retinopathy. Most FGFRi-associated retinopathy were Grade 1/2 events. Grade 3 FGFRi-associated retinopathy were infrequent (mUC pooled studies, 11/479 [2.3%]; RAGNAR, 3/314 [1.0%]). No Grade 4 events occurred. Most FGFRi-associated retinopathy events (mUC pooled studies, 81/103 [78.6%]; RAGNAR, 30/43 [70.0%]) occurred within 90 days of treatment initiation and were managed with erdafitinib dose interruption (mUC pooled studies, 41/479 [8.6%]; RAGNAR, 23/314 [7.3%]) or reduction (mUC pooled studies, 58/479 [12.1%]; RAGNAR, 21/314 [6.7%]); few required treatment discontinuation (mUC pooled studies, 14/479 [2.9%]; RAGNAR, 3/314 [1.0%]). By the data cutoff, 63.1% (65/103) patients in the mUC pooled studies and 65.1% (28/43) patients in RAGNAR had their FGFRi-associated retinopathy events resolve. Most unresolved FGFRi-associated retinopathy events were persistent Grade 1 events. In RAGNAR, 92.0% of patients had their visual acuity return to baseline and 78.5% of patients had their retinal pigment epithelial elevation resolve.

**Conclusions:**

FGFRi-associated retinopathy with erdafitinib can be managed with dose reductions, interruptions, or discontinuations. Proactive collaboration between oncologists and ophthalmologists is required to ensure that erdafitinib delivers clinical benefit to patients while managing their potential adverse symptoms of FGFRi-associated retinopathy.

Implications for Practice Fibroblast growth factor receptor inhibitor (FGFRi)-associated retinopathy is a class effect of FGFRi, including erdafitinib. Approximately 15%–20% of patients across 6 clinical studies of metastatic urothelial cancer or other advanced solid tumors treated with erdafitinib experienced FGFRi-associated retinopathy. Most FGFRi-associated retinopathy were Grade 1/2 events. The majority of events occurred within the first 90 days of erdafitinib treatment and were effectively managed with erdafitinib dose reductions, interruptions, or discontinuations. Most unresolved FGFRi-associated retinopathy were persistent Grade 1 events. Effective management of FGFRi-associated retinopathy with erdafitinib requires proactive collaboration between ophthalmologists and oncologists to ensure that erdafitinib delivers clinical benefit to patients while managing their potential FGFRi-associated retinopathy.

## Introduction

Fibroblast growth factors receptor inhibitors (FGFRi) are an emerging class of targeted cancer therapies with clinical relevance for the treatment of a variety of malignancies with *FGFR* alterations, including urothelial carcinoma and cholangiocarcinoma.^1–4^ Since FGFRi disrupt key cellular pathways such as tumor cell proliferation, survival, migration, and angiogenesis by blocking the FGFR pathway, their class effects may result in a variety of adverse events, including ocular adverse events such as FGFRi-associated retinopathy.^5–8^ FGFRi-associated retinopathy has been reported at varying frequencies in patients treated with FGFRi, including erdafitinib, pemigatinib, infigratinib, and rogaratinib.^6^^,^^7^

Since the FGFR pathway is known to intersect with downstream mitogen-activated protein kinase (MAPK) signaling, a pathway involved in maintenance, repair, and survival of retinal pigment epithelial cells, the pathophysiology of FGFRi-associated retinopathy resembles that of retinopathy associated with the use of MAPK and extracellular signal-regulated kinase inhibitors.^5–8^ Although poorly understood, this pathophysiology is characterized by accumulation of subretinal fluid between the retinal pigment epithelium and interdigitation zone of the photoreceptor layer; as such, the hyperreflective photoreceptor and retinal pigment epithelial layers remain intact, without retinal pigmental epithelial detachment or choroidal thickening.^7^^,^^9^ This pathophysiology is distinct from central serous retinopathy and diabetic retinopathy.^7^^,^^9^^,^^10^ In contrast to FGFRi-associated retinopathy, central serous retinopathy and diabetic retinopathy are associated with permanent visual impairment.^10^^,^^11^ FGFRi-associated retinopathy may be detected with or without routine optical coherence tomography testing and may manifest through a variety of ocular symptoms, including blurred vision, metamorphopsias, and scotoma^6–8^; however, these symptoms may be mild or absent.^6–9^ Although FGFRi-associated retinopathy is not associated with significant long-term vision loss and is self-limiting or reversible,^6–9^ these events may lead to treatment reductions, interruptions, or discontinuations.^7^^,^^9^

Erdafitinib, an oral, pan-FGFR tyrosine kinase inhibitor, is indicated for the treatment of adult patients with locally advanced or metastatic urothelial carcinoma (mUC) with susceptible *FGFR3* genetic alterations, whose disease has progressed on or after at least one line of programmed cell death protein-(ligand)1 [PD-(L)1] inhibitor therapy.^12^^,^^13^ Herein, we summarize FGFRi-associated retinopathy using safety data across the oral erdafitinib clinical trial development program.

## Patients and methods

### Overview of studies

This analysis summarizes the incidence, time to onset, clinical course, and management of FGFRi-associated retinopathy (including modifications to erdafitinib treatment), and baseline characteristics of patients with FGFRi-associated retinopathy across 6 clinical studies of oral erdafitinib. This analysis pools together data from 5 clinical studies (hereafter referred to as the mUC pooled studies) in which adult patients with locally advanced or mUC received 8 or 9 mg oral erdafitinib: THOR,^14^^,^^15^ BLC2001,^3^ BLC2001 drug-drug interaction (DDI) substudy,^16^ NORSE,^17^ and EDI1001.^18^ In addition, this analysis presents separate results from the RAGNAR study in patients with advanced solid tumors other than mUC.^19^^,^^20^ All studies excluded patients with a history of or current evidence of FGFRi-associated retinopathy. SAS version 9.4 was used for all statistical analyses.

### mUC pooled studies

#### THOR (NCT03390504)

THOR was a phase III, randomized, controlled, open-label study conducted in 2 cohorts of patients with mUC harboring susceptible *FGFR3/2* alterations. In Cohort 1, patients with mUC who had prior anti-PD-(L)1 therapy were randomly assigned 1:1 to erdafitinib 8 mg once daily or chemotherapy (vinflunine or docetaxel).^14^ In Cohort 2, patients with mUC naive to anti-PD-(L)1 therapy who had one prior line of therapy were randomly assigned 1:1 to erdafitinib 8 mg once daily or pembrolizumab.^15^ In both cohorts, erdafitinib could be uptitrated to 9 mg based on the patient’s serum phosphate levels.^14^^,^^15^ Among 593 patients treated in THOR Cohorts 1 and 2 at the time of primary analysis, the 308 patients who were treated with erdafitinib are included in the pooled analysis.

#### BLC2001 (NCT02365597)

BLC2001 was a phase II, open-label study that investigated 3 erdafitinib dosing regimens in patients with mUC harboring 1 or more *FGFR3/2* alterations: 10 mg intermittent (7 days on/7 days off), 6 mg daily, or 8 mg once daily with potential uptitration to 9 mg.^3^ Among 212 patients treated across dosing regimens in BLC2001, the 101 patients who received the erdafitinib 8 mg once daily dosing regimen are included in the pooled analysis.

#### BLC2001 DDI substudy

The BLC2001 DDI substudy evaluated the effects of repeated erdafitinib dosing on the single-dose pharmacokinetics of midazolam (sensitive CYP3A4 substrate) and metformin (sensitive OCT2 substrate) in patients with malignant, advanced solid tumors harboring select *FGFR3/2* alterations. Erdafitinib was administered 8 mg once daily on Days 1–12. Erdafitinib was then co-administered with single doses of either midazolam 2.5 mg on Day 13, or metformin 1000 mg on Day 14, with potential uptitration to 9 mg on Day 15 depending on serum phosphate levels measured on Day 14.^16^ Among 25 patients treated in the BLC2001 DDI substudy, 15 patients with mUC are included in the pooled analysis.

#### NORSE (NCT03473743)

NORSE was a phase Ib/II, open-label study in patients with mUC harboring select *FGFR3/2* alterations. In dose expansion, patients were randomly assigned 1:1 to receive either erdafitinib 8 mg once daily with pharmacodynamically guided uptitration to 9 mg (Arm A) or erdafitinib 8 mg once daily (no uptitration) plus intravenous cetrelimab 240 mg every 2 weeks (Arm B).^17^ Among 87 patients treated in dose expansion, the 43 patients treated with erdafitinib 8 mg with pharmacodynamically guided uptitration to 9 mg are included in the pooled analysis.

#### EDI1001 (NCT01703481)

EDI1001 was a phase I, first-in-human, open-label study in patients with malignant solid tumors or lymphoma. Erdafitinib was administered either once daily at ≤4, 6, 9, or 12 mg, or intermittently (7 days on/7 days off) at 10 or 12 mg.^18^ Among 187 patients treated, the 12 patients with mUC who received the erdafitinib 9 mg once-daily dosing regimen are included in the pooled analysis.

#### RAGNAR (NCT04083976; EudraCT 2019-002113-19)

RAGNAR was a phase II, open-label, tumor-agnostic study in patients with advanced solid tumors who were enrolled in 4 cohorts based on tumor histology, *FGFR* alteration type, and age. Adults and adolescents ≥15 to <18 years old received erdafitinib 8 mg once daily with potential uptitration to 9 mg. Adolescents ≥12 to <15 years old received erdafitinib 5 mg once daily with potential uptitration to 6–8 mg. Children ≥6 to <12 years old received erdafitinib 3 mg once daily with potential uptitration to 4–5 mg.^19^^,^^20^ Data from the 314 patients ≥6 years old with advanced solid tumors harboring select *FGFR* gene alterations treated with erdafitinib are presented separately from the mUC pooled studies in this analysis.

### Characterization of FGFRi-associated retinopathy events

FGFRi-associated retinopathy represents a group of treatment-emergent adverse events (TEAEs) reported across the oral erdafitinib clinical trials. These TEAEs included diagnoses such as detachment of retinal pigment epithelium, serous retinopathy, subretinal fluid, retinal edema, retinal thickening, chorioretinopathy, choroidal effusion, macular detachment, maculopathy, retinal detachment, retinopathy, serous retinal detachment, chorioretinitis, and vitreous detachment.

Grade 1–4 FGFRi-associated retinopathy was defined based on the following signs, symptoms, and severity according to the National Cancer Institute Common Terminology Criteria for Adverse Events: Grade 1 = asymptomatic or mild symptoms by clinical or diagnostic observations only or positive Amsler grid test; Grade 2 = moderate symptoms that potentially limit age-appropriate instrumental activities of daily living with local or noninvasive intervention indicated, and visual acuity 20/40 or better or ≤3 lines of decreased vision from baseline; Grade 3 = severe or medically significant symptoms that limit self-care activities of daily living but are not immediately sight-threatening, with hospitalization or prolongation of existing hospitalization indicated and visual acuity worse than 20/40 or >3 lines of decreased vision from baseline; Grade 4 = blindness (20/200 or worse) in the affected eye with urgent intervention indicated.

For all patients who reported a FGFRi-associated retinopathy: if multiple reports of the same TEAE were reported within 3 days of one another, the events were considered the same event. For patients whose FGFRi-associated retinopathy resolved, the median time to resolution was determined for events that occurred in 3 or more patients.

For patients with unresolved FGFRi-associated retinopathy, the event was defined as follows:

- If the toxicity grade did not change from Grade 1, this was considered “Grade 1 persistent toxicity.”
- If the toxicity grade improved from (1) Grade 3 to Grade 2 or (2) remained at Grade 2, this was considered “unresolved Grade 2.”
- If the toxicity grade of the event remained at Grade 3, this was considered “unresolved Grade 3.”
- If toxicity grade improved from Grade 2 or 3 to Grade 1, this was considered “a higher grade that improved to Grade 1.”

### Ophthalmologic examinations

At screening, all patients received an ophthalmologic examination performed by an ophthalmologist. Patients treated with erdafitinib received an Amsler grid test at screening, on Day 1 of every treatment cycle beginning at Cycle 2, and at their end-of-treatment visit. The Amsler grid—a 10 cm × 10 cm square white paper displaying vertical and horizontal lines that form 20x20 cells and a black dot in the center—is used to identify visual distortions. A positive, abnormal, Amsler grid test is defined as seeing (1) wavy, broken, or distorted lines or (2) a blurred/missing area of vision when looking at the central dot.^21^ If positive results were found by Amsler grid test or if patient-reported symptoms indicated a FGFRi-associated retinopathy event (such as blurred vision, partial/complete loss of vision, double vision, floaters/color spots/halos around light, changes in color/night vision, photophobia, or ocular pain) at any time, then the study oncologist referred the patient to an ophthalmologist for follow-up ophthalmologic examination and optical coherence tomography.

### Visual acuity and optical coherence tomography imaging assessments

Patients had their visual acuity tested and reported separately for each eye under the direction of an ophthalmologist. Patient visual acuity data were summarized by the Snellen format; data from different reporting methods that could not be converted to Snellen were excluded from this analysis. All patients received baseline ophthalmologic examinations per protocol. Optical coherence tomography examinations were interpreted by an ophthalmologist.

Optical coherence tomography included assessments of the patient’s subretinal fluid and retinal pigment epithelial elevations, which are thought to best represent the presence of fluid accumulation between the retinal pigment epithelium and Bruch’s membrane,^22^ a characteristic of the grouped FGFRi-associated retinopathy term. Patients received post-baseline ophthalmologic examinations only when deemed clinically necessary based on Amsler grid tests and clinical assessment, or at regular intervals as deemed necessary by the screening ophthalmologist. In addition in the RAGNAR study, patients who had a baseline and at least 1 post-baseline optical coherence tomography had their subretinal fluid and retinal pigment epithelial elevation fluid assessed by an independent blinded assessor.

### Management of FGFRi-associated retinopathy events across the oral erdafitinib clinical studies

In the oral erdafitinib clinical studies, patients with a positive Amsler grid test and/or symptoms indicative of any grade/severity and type of FGFRi-associated retinopathy were referred to an ophthalmologist for evaluation. Appropriate management of FGFRi-associated retinopathy was then guided by the severity and clinical course of the FGFRi-associated retinopathy event (Table S1). Recommended FGFRi-associated retinopathy management included in the erdafitinib prescribing information is generally consistent with the approach used across the oral erdafitinib clinical studies.^12^^,^^13^

## Results

### mUC pooled studies

**Figure 1.**
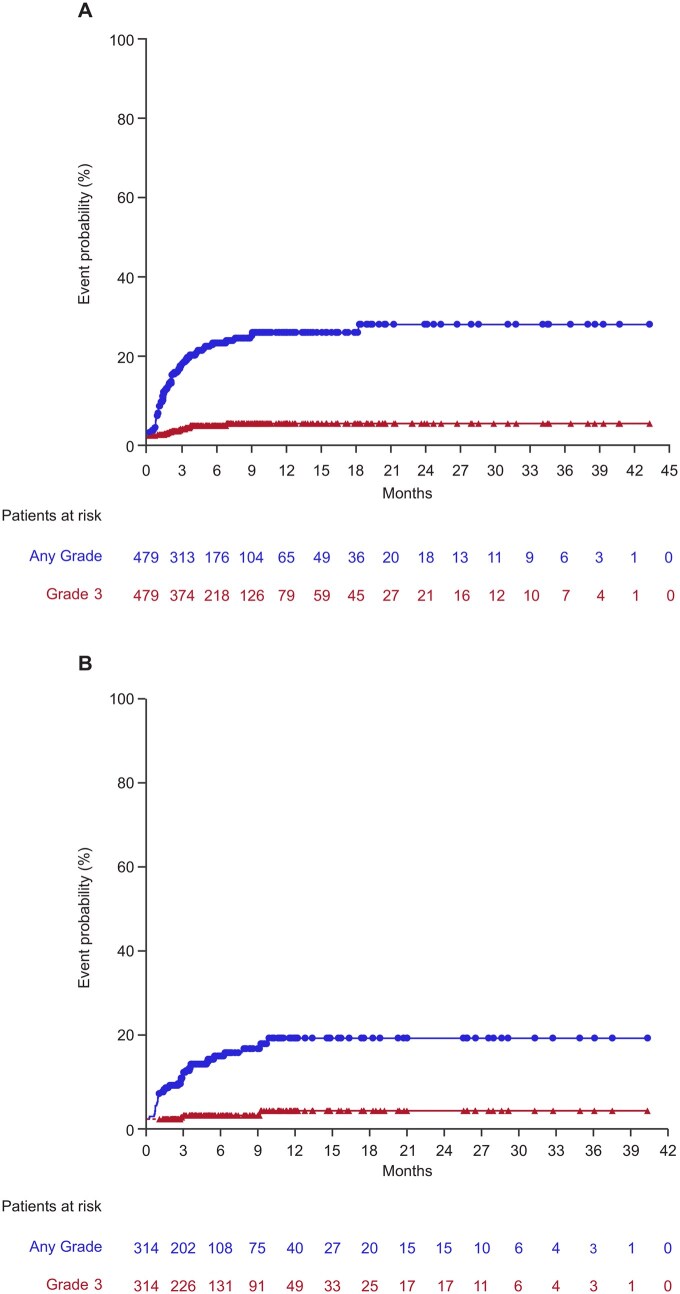
**Kaplan-Meier plot of time to first onset of FGFRi-associated retinopathy in the mUC pooled studies (A) and the RAGNAR study (B)**. Patients without corresponding events are censored at date of last dose +30 days, data cutoff, or the end of study date, whichever is first. Abbreviations: mUC, metastatic urothelial cancer.

Among 479 patients included in the mUC pooled studies, 103 (21.5%) experienced FGFRi-associated retinopathy (Table 1). Patients ≥65 years old had higher FGFRi-associated retinopathy incidence than patients <65 years old (73/288 [25.3%] vs 30/191 [15.7%]). The most common (>2%) FGFRi-associated retinopathy events were chorioretinopathy, retinal pigment epithelium detachment, retinal detachment, retinopathy, and subretinal fluid (Table 1). Patients ≥65 years old more frequently reported retinal pigmental epithelial detachment than patients <65 years old (18 [6.3%] vs 4 [2.1%]). Most FGFRi-associated retinopathy were Grade 1 or 2 (92/479 [19.2%]), 11/479 (2.3%) patients experienced Grade 3 events, and no Grade 4 events occurred (Table 2). Among the 103 patients who had FGFRi-associated retinopathy, the majority (81 [78.6%]) had events that occurred within the first 90 days of erdafitinib treatment (Figure 1A). Median time to first onset was 45 (range, 5–554) days for any-grade FGFRi-associated retinopathy and 83 (range, 24–207) days for Grade 3 FGFRi-associated retinopathy (Table 2).

**Table 1.** Incidence of FGFRi-associated retinopathy in RAGNAR and the mUC pooled clinical studies.

	mUC pooled studies (*N* = 479)	RAGNAR (*N* = 314)
**FGFRi-associated retinopathy incidence**	103 (21.5)	43 (13.7)
**FGFRi-associated retinopathy preferred term**		
** Chorioretinopathy**	30 (6.3)	12 (3.8)
** Retinal pigment epithelium detachment**	22 (4.6)	8 (2.5)
** Retinal detachment**	12 (2.5)	7 (2.2)
** Retinopathy**	11 (2.3)	5 (1.6)
** Subretinal fluid**	10 (2.1)	5 (1.6)
** Serous retinal detachment**	8 (1.7)	5 (1.6)
** Vitreous detachment**	7 (1.5)	1 (0.3)
** Maculopathy**	4 (0.8)	2 (0.6)
** Retinal edema**	3 (0.6)	4 (1.3)
** Macular retinal pigment epithelium detachment**	2 (0.4)	0
** Macular detachment**	2 (0.4)	2 (0.6)
** Chorioretinitis**	1 (0.2)	0
** Retinal thickening**	1 (0.2)	2 (0.6)
** Serous retinopathy**	1 (0.2)	2 (0.6)

Data are reported as *n* (%).

Abbreviations: FGFRi, fibroblast growth factor receptor inhibitor; mUC, metastatic urothelial cancer.

**Table 2.** Characterization of FGFRi-associated retinopathy in RAGNAR and the mUC pooled clinical studies.

	mUC pooled studies (*N* = 479)	RAGNAR (*N* = 314)
**FGFRi-associated retinopathy incidence**	103 (21.5)	43 (13.7)
** Maximum severity of event**		
** Grade 1 or 2**	92 (19.2)	40 (12.7)
** Grade 3**	11 (2.3)	3 (1.0)
** Serious events**	12 (2.5)	3 (1.0)
** Patients with events requiring erdafitinib dose modification^a^**		
** Reduction**	58 (12.1)	23 (7.3)
** Interruption**	41 (8.6)	21 (6.7)
** Patients with events requiring erdafitinib dose discontinuation**	14 (2.9)	3 (1.0)
** Time to first event onset (days), median (range)**		
** Any-grade events**	45 (5–554)	44 (8–297)
** Grade 3 events**	83 (24–207)	92 (85–280)
** Patients with event onset after erdafitinib initiation^b,c^**	*n* = 103	*n *= 43
** ≤1 month**	34 (33.0)	17 (39.5)
** >1–2 months**	27 (26.2)	8 (18.6)
** >2–3 months**	20 (19.4)	5 (11.6)
** >3 months**	22 (21.4)	13 (30.2)
** Event resolution status at data cutoff^c^**	*n* = 103	*n* = 43
** Resolved**	65 (63.1)	28 (65.1)
** Unresolved**	38 (36.9)	15 (34.9)
** Grade 1**	23 (22.3)	12 (27.9)
** Grade 2**	12 (11.7)	2 (4.7)
** Grade 3**	3 (2.9)	1 (2.3)

Data are reported as *n* (%).

a Dose modification was reported as either an interruption or reduction but not both, so patients who had a dose reduction may have also had a preceding interruption related to the same event.

b Only patients who were treated the specified number of months after erdafitinib initiation and only FGFRi-associated retinopathy that occurred during that time period were counted. FGFRi-associated retinopathy events occurring beyond the last erdafitinib dose were assigned to the last time period in which a patient was treated.

c Percentage calculations are based on overall incidence of FGFRi-associated retinopathy as the denominator.

Abbreviations: FGFRi, fibroblast growth factor receptor inhibitor; mUC, metastatic urothelial cancer.

FGFRi-associated retinopathy led to erdafitinib dose interruption in 41/479 (8.6%) patients and dose reduction in 58/479 (12.1%) patients (Table 2). Erdafitinib dose interruptions and reductions due to FGFRi-associated retinopathy events were more common in patients ≥65 years old than patients <65 years old (dose interruptions, 32/288 [11.1%] vs 9/191 [4.7%]; dose reductions, 41/288 [14.2%] vs 17/191 [8.9%]). Overall, 14/479 (2.9%) patients discontinued erdafitinib due to 1 or more of these FGFRi-associated retinopathy events: retinal pigment epithelium detachment (8 [1.7%]), chorioretinopathy (3 [0.6%]), maculopathy (2 [0.4%]), retinal detachment (1 [0.2%]), and subretinal fluid (1 [0.2%]). FGFRi-associated retinopathy leading to erdafitinib discontinuation were more common in patients ≥65 years old than in patients <65 years old (13/288 [4.5%] vs 1/191 [0.5%]) and included retinal pigment epithelial detachment (*n* = 7), chorioretinopathy (*n* = 3), maculopathy (*n* = 2), retinal detachment (*n* = 1), and subretinal fluid (*n* = 1).

The majority (63.1% [65/103]) of patients had their FGFRi-associated retinopathy resolve (Table 2). Resolution rate of FGFRi-associated retinopathy at data cutoff was lower in patients ≥65 years old than patients <65 years old (44/73 [60.3%] vs 21/30 [70.0%]). Median time to resolution ranged from 21 days for subretinal fluid (*n* = 7) to 97 days for vitreous detachment (*n* = 3). Chorioretinopathy, the most common FGFRi-associated retinopathy, resolved in 22 of 30 patients within a median of 24 days. Time to resolution of FGFRi-associated retinopathy appeared to increase with toxicity grade.

FGFRi-associated retinopathy remained unresolved in 36.9% (38/103) of patients at the time of data cutoff (Table 2). The majority of unresolved FGFRi-associated retinopathy were Grade 1 (in 60.5% [23/38] patients), including 15 patients with persistent Grade 1 events and 8 patients with Grade 2 or 3 events that improved to Grade 1. Additionally, 12 patients had unresolved Grade 2 events (chorioretinopathy, retinal pigment epithelial detachment, *n* = 6 each), and 3 patients had unresolved Grade 3 events (retinal pigment epithelial detachment, *n* = 2; maculopathy, *n* = 1).

#### RAGNAR

In 314 patients with advanced solid tumors other than mUC treated in RAGNAR, 43 (13.7%) patients experienced FGFRi-associated retinopathy (Table 1). Incidence of FGFRi-associated retinopathy was similar by patient’s age (>65 years, 11/79 [13.9%] vs ≤65 years, 32/235 [13.6%]), sex (male, 24/155 [15.5%] vs female, 19/159 [11.9%]), and baseline creatinine clearance (<60 mL/min, 3/33 [9.1%] vs ≥60 mL/min, 40/281 [14.2%]). Most FGFRi-associated retinopathy events were Grade 1 or 2 (Table 2). Three (1.0%) patients had Grade 3 FGFRi-associated retinopathy: chorioretinopathy, retinal pigment epithelial detachment, and retinal edema (*n* = 1 each). No patients had Grade 4 FGFRi-associated retinopathy. The median time to FGFRi-associated retinopathy onset was 44 (range, 8–297) days for any-grade events and 92 (range, 85–280) days for Grade 3 events (Table 2). Most FGFRi-associated retinopathy events occurred within the first 90 days of erdafitinib treatment (in 30/43 [69.8%] patients; Figure 1B). FGFRi-associated retinopathy led to erdafitinib dose interruption in 21/314 (6.7%) patients and dose reduction in 23/314 (7.3%) patients (Table 2). Three (1.0%) of 314 patients discontinued erdafitinib due to FGFRi-associated retinopathy: maculopathy, retinopathy, and serous retinal detachment (*n* = 1 each).

The majority (65.1% [28/43]) of patients had their FGFRi-associated retinopathy resolve by the time of data cutoff (Table 2). Median time to resolution ranged from 14 days (retinal edema, *n* = 3) to 126 days (retinal pigmental epithelium detachment, *n* = 5). The most common FGFRi-associated retinopathy event of chorioretinopathy resolved in 9 of 12 patients within a median of 16 days.

FGFRi-associated retinopathy remained unresolved in 34.9% (15/43) of patients. Most unresolved FGFRi-associated retinopathy events were Grade 1 (in 80.0% [12/15] patients), including 11 patients with persistent Grade 1 events, and 1 patient with a Grade 2 event that improved to Grade 1. Additionally, 2 patients had unresolved Grade 2 events (retinopathy, *n* = 1; maculopathy, *n* = 1) and 1 patient had unresolved Grade 3 retinal pigmental epithelium detachment.

### Visual acuity and optical coherence tomography assessments in RAGNAR

**Figure 2.**
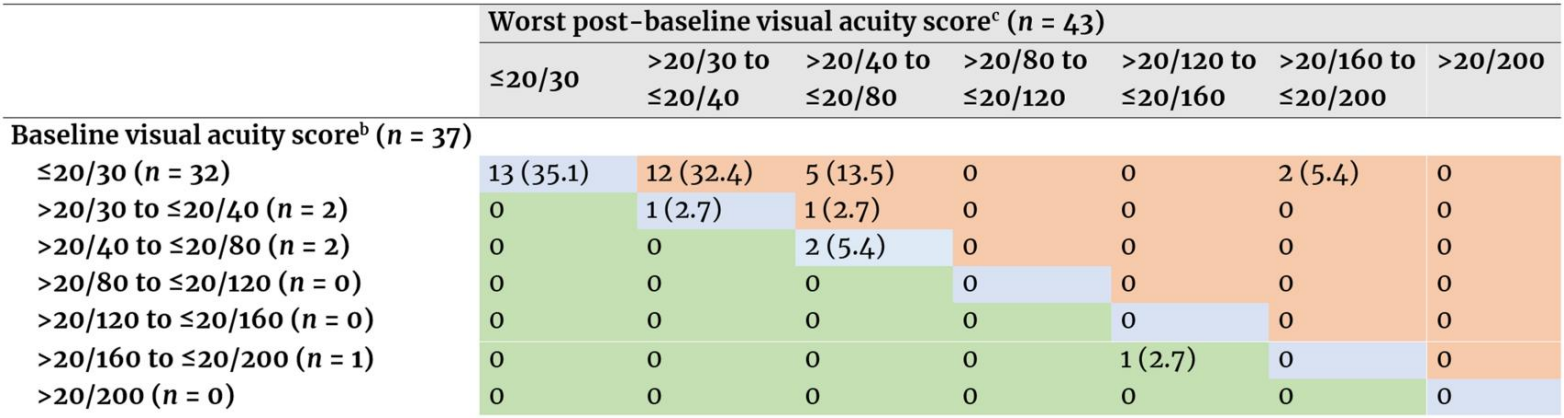
**Changes in visual acuity from baseline to worst post-baseline assessment in patients with FGFRi-associated retinopathy^a^ in RAGNAR**. Relative to baseline, green shading indicates improvement, blue shading indicates no change, and orange shading indicates worsening. Patients’ visual acuity results were measured with the Snellen chart and the Landolt ring chart. *n* is the number of patients who had non-missing values for visual acuity at both baseline and at least 1 post-baseline visit that were convertible to the Snellen format. ^a^ FGFRi-associated retinopathy was defined as retinal detachment, vitreous detachment, retinal edema, retinopathy, chorioretinopathy, retinal pigment epithelium detachment, or macular retinal pigment epithelium detachment. ^b^ Baseline visual acuity was the baseline value for whichever eye was reported as the worst post-baseline value. Abbreviations: FGFRi, fibroblast growth factor receptor inhibitor. ^c^ Worst post-baseline visual acuity was defined as the value that resulted in the largest change from baseline value for either eye.

**Figure 3.**
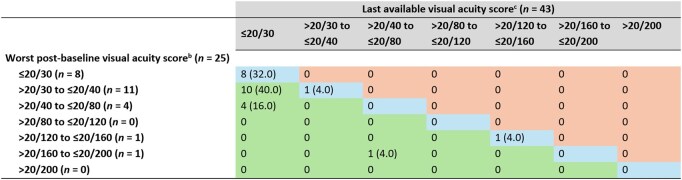
**Changes in visual acuity from worst post-baseline assessment to the last available assessment in patients with FGFRi-associated retinopathy^a^ in RAGNAR.** Relative to the worst post-baseline assessment, green shading indicates improvement, blue shading indicates no change, and orange shading indicates worsening. Patients’ visual acuity results were measured with the Snellen chart and the Landolt ring chart. *n* is the number of patients who had non-missing values for visual acuity with at least 1 visit following the first occurrence of the worst post-baseline visit. ^a^ FGFRi-associated retinopathy was defined as retinal detachment, vitreous detachment, retinal edema, retinopathy, chorioretinopathy, retinal pigment epithelium detachment, or macular retinal pigment epithelium detachment. ^b^ Worst post-baseline visual acuity was defined as the value that resulted in the largest change from baseline value for either eye. ^c^ Last available visual acuity was the last available value for whichever eye is reported as the worst post-baseline value. Abbreviations: FGFRi, fibroblast growth factor receptor inhibitor.

**Figure 4.**
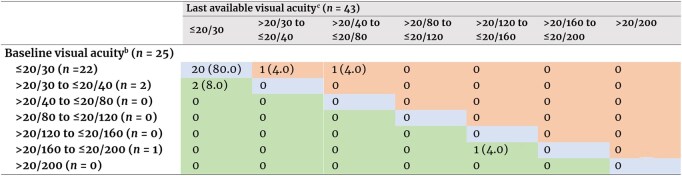
**Changes in visual acuity from baseline to last available assessment in patients with FGFRi-associated retinopathy^a^ in RAGNAR.** Relative to baseline, green shading indicates improvement, blue shading indicates no change, and orange shading indicates worsening. Patients’ visual acuity results were measured with the Snellen chart and the Landolt ring chart. *n* is the number of patients who had non-missing values for visual acuity with at least 1 visit following the first occurrence of the worst post-baseline visit. ^a^ FGFRi-associated retinopathy was defined as retinal detachment, vitreous detachment, retinal edema, retinopathy, chorioretinopathy, retinal pigment epithelium detachment, or macular retinal pigment epithelium detachment. ^b^ Baseline visual acuity was the baseline value for whichever eye is reported as the worst post-baseline value. ^c^ Last available visual acuity was the last available value for whichever eye is reported as the worst post-baseline value. Abbreviations: FGFRi, fibroblast growth factor receptor inhibitor.

Among 43 patients who experienced FGFRi-associated retinopathy, 37 had both a baseline and at least 1 post-baseline visual acuity assessment converted to the Snellen format. Among these patients, 20/37 (54.1%) experienced a decrease in visual acuity from baseline to the worst post-baseline assessment. Among 32 patients with baseline visual acuity ≤20/30 who had FGFRi-associated retinopathy, 19 (59.4%) patients experienced worse visual acuity following erdafitinib treatment, including 2 patients whose visual acuity decreased from >20/160 to ≤20/200 as their worst post-baseline assessment (Figure 2). Among 25 patients who had both a baseline and subsequent visual acuity assessment following the worst post-baseline visual acuity assessment, 23 (92.0%) patients returned to baseline visual acuity or better at the last available assessment (Figure 3). At the last available visual acuity assessment, 2 patients with visual acuity ≤20/30 experienced modest worsening (Figure 4).

Thirty-six patients with FGFRi-associated retinopathy and both a baseline and at least 1 post-baseline optical coherence tomography had retinal pigmental epithelium assessment; among these, 13 (36.1%) patients had post-baseline retinal pigmental epithelium elevation. Among 28 patients further assessed for retinal pigmental epithelium after their worst post-baseline retinal pigmental epithelium elevation, 22 (78.6%) patients were stable or resolved relative to baseline and 6 (21.4%) patients had increased retinal pigmental epithelium elevation relative to baseline considered unresolved at the time of the last available assessment.

Thirty-seven patients who experienced FGFRi-associated retinopathy and had both a baseline and at least 1 post-baseline optical coherence tomography had their subretinal fluid assessed by optical coherence tomography; 35 patients had no subretinal fluid and 2 patients had subretinal fluid at baseline. Among 35 patients who had no subretinal fluid at baseline, 34 (97.1%) patients had increased subretinal fluid and 1 (2.9%) patient had stable subretinal fluid from baseline as their worst post-baseline assessment; at their last available assessment, 19 (54.3%) patients had stable/resolved subretinal fluid and 16 (45.7%) patients had subretinal fluid that was considered not fully resolved compared with their worst post-baseline assessment. Among 2 patients who had subretinal fluid at baseline, 1 patient had increased subretinal fluid and 1 patient had stable subretinal fluid as their worst post-baseline assessment.

## Discussion

Retinal toxicity is a known class effect of FGFRi. Erdafitinib is approved for adult patients with previously treated mUC and susceptible *FGFR3* genetic alterations.^12^^,^^13^ Other treatments such as pan-FGFR (futibatinib) and FGFR1/2/3 inhibitors (infigratinib, pemigatinib) are approved for non urothelial cancers.^23–25^ Diagnosis and monitoring of the incidence, severity, clinical manifestations, and progression of drug-related retinal toxicities along with appropriate management strategies are essential for supporting informed clinical decision-making while improving clinical outcomes for patients taking FGFRi.

To our knowledge, this report represents the largest body of data on FGFRi-associated retinopathy published to date. In our pooled analysis, the incidence of FGFRi-associated retinopathy ranged from 14% to 22% across 6 clinical studies of oral erdafitinib in patients with mUC or other advanced solid tumors. The frequency of erdafitinib-associated retinopathy observed in our analysis appears generally consistent with a published retrospective analyses of FGFRi, in which FGFRi-associated retinopathy was reported in 13.7% of patients; retinal pigment epithelial detachment has also been observed with commercially available therapies such as pemigatinib (11%) and futibatinib (7.8%).^6^^,^^23^^,^^24^ However, direct cross-study comparisons are limited by differences in ophthalmologic surveillance protocols, such as frequency of assessments, use of optical coherence tomography, categorization of FGFRi-associated retinopathy events, and patient populations.

Most FGFRi-associated retinopathy events observed in this study occurred during the first 3 months of erdafitinib treatment. Importantly, the majority of FGFRi-associated retinopathy events were Grade 1 or 2. Grade 3 events were infrequent, and no patient experienced blindness (Grade 4 event). Across RAGNAR and the mUC pooled studies, over 60% of patients had their FGFRi-associated retinopathy resolve by the data cutoff. For patients with unresolved FGFRi-associated retinopathy by data cutoff, most had persistent Grade 1 events. FGFRi-associated retinopathy rarely led to erdafitinib discontinuation and was manageable with dose modifications (dose interruption and/or reduction). Further, visual acuity and optical coherence tomography imaging assessments in the RAGNAR clinical study demonstrated that most patients’ visual acuity and retinal pigment epithelial elevation either returned to baseline or stabilized by their last assessment.

Subgroup analyses by patient age in the mUC pooled studies suggest that FGFRi-associated retinopathy events may be more common in patients older than 65 years, highlighting the importance of increased vigilance in older patients. This trend was not observed in the RAGNAR study, possibly due to a lower proportion of older patients enrolled in the RAGNAR study (25% of patients were >65 years old) compared with the patient population enrolled in the mUC pooled studies (60% of patients were ≥65 years old). The small number of patients who experienced FGFRi-associated retinopathy in RAGNAR limits our ability to determine the impact of baseline characteristics (eg, age, sex, and creatinine levels) on patients’ optical coherence tomography measures, such as subretinal fluid and retinal pigment epithelial elevation.

This analysis should be interpreted in the context of the following limitations. End-of-data collection at data cutoff may have led to overestimation of unresolved FGFRi-associated retinopathy. In addition, while optical coherence tomography scans were performed per study protocols, scan readings by an independent blinded assessor for the mUC studies were not available at the time of our analysis; this raises the possibility that the same FGFRi-associated retinopathy may be described by patients using different terms, which could lead to potential overlap in how FGFRi-associated retinopathy events were characterized. Our analysis population comprised a selected sample of patients with cancer based on clinical study eligibility criteria; caution is recommended when extrapolating these findings to patients in the real world who may have significant comorbidities that would exclude them from clinical studies. Finally, it is important to highlight that based on protocol-provided guidance, erdafitinib treatment was interrupted for patients who experienced FGFRi-associated retinopathy of any grade; this conservative approach did not allow for exploring the possibility that some of these FGFRi-associated retinopathy events may have been self-limiting and may not have required erdafitinib interruption in some cases. Resolution of ocular findings related to FGFRi-associated retinopathy without medical intervention or FGFRi interruption has been reported in the literature.^6^

Based on available data, the following guidelines are currently recommended in the erdafitinib product label: (1) monthly ophthalmological examinations including visual acuity, slit lamp, fundoscopy, and optical coherence tomography during the first 4 months of treatment, every 3 months thereafter, and at any time for visual symptoms; and (2) erdafitinib should be withheld when FGFRi-associated retinopathy occurs and should be permanently discontinued if no resolution is observed within 4 weeks or if there are sight-threatening consequences or blindness.^12^^,^^13^ A multidisciplinary approach with close collaboration between oncologists and ophthalmologists is strongly recommended to allow for personalized management decisions based on ophthalmologic findings and to optimize clinical outcomes in patients taking erdafitinib, considering the impact of withholding treatment on the patient’s cancer course and clinical outcomes.^9^

## Summary

In summary, the overall benefit-risk profile of erdafitinib for the approved indication of mUC is favorable. However, proactive collaboration between oncologists and ophthalmologists is necessary to ensure that erdafitinib provides clinical benefit to patients with cancer while managing the potential adverse symptoms of FGFRi-associated retinopathy.

## Supplementary Material

oyag174_Supplementary_Data

## Data Availability

The data sharing policy of Johnson & Johnson is available at https://innovativemedicine.jnj.com/our-innovation/clinical-trials/transparency. As noted on this site, requests for access to the study data can be submitted through the Yale Open Data Access (YODA) Project site at http://yoda.yale.edu.
