# Supplementary material for: Incidence and outcomes of FGFR inhibitor-associated retinopathy of patients treated with oral erdafitinib across the clinical trial program
Source: Oncologist. 2026 May 13;31(7):oyag174. doi: 10.1093/oncolo/oyag174 (PMC13293072; doi:10.1093/oncolo/oyag174)
Supplement: oyag174_Supplementary_Data [file oyag174_supplementary_data.zip › 22-May-2026_122541_OHagan_The_Oncologist_SupplementaryMaterial_17APR2026.docx]

**Incidence and outcomes of FGFR inhibitor-associated retinopathy of patients treated with oral erdafitinib across the clinical trial program**

Anne O’Hagan, Arlene Siefker-Radtke, Yohann Loriot, Kris Deprince, Lauren Crow,
Michal Laron, Ron Adelman, Hussein Sweiti, Spyros Triantos

Supplementary Material

Supplementary Table S1. Management of FGFR inhibitor-associated retinopathy used across oral erdafitinib clinical studies

| **Severity and grading** | **Immediate action** | **Dose management** |
| --- | --- | --- |
| **Grade 1**  Asymptomatic or mild symptoms; clinical or diagnostic observations only, or abnormal Amsler grid test | Refer to an ophthalmologist  If an ophthalmologist evaluation was not performed within 7 days, erdafitinib was immediately withheld until an ophthalmologist evaluation was performed | If diagnosis from ophthalmologist evaluation was retinal abnormality, erdafitinib was withheld until signs, symptoms, and ocular findings were resolved  If signs and symptoms resolved within 4 weeks of ophthalmologist evaluation, erdafitinib was resumed at the next lower dose level  If no recurrence was observed at this dose level after 1 month, then erdafitinib dose re-escalation was considered |
| **Grade 2**  Moderate; limiting age-appropriate instrumental activities of daily living | Refer for an ophthalmologist evaluation  Immediately withhold erdafitinib | If there was no evidence of FGFRi-associated retinopathy, erdafitinib was continued at the next lower dose level upon resolution  If signs, symptoms, and ocular findings resolved within 4 weeks of ophthalmologist evaluation, then erdafitinib was resumed at the next lower dose level  This dose level was monitored for recurrence |
| **Grade 3**  Severe or medically significant but not immediate sight threatening; limiting self-care activities of daily living | Refer for an ophthalmologist evaluation  Immediately withhold erdafitinib | If symptoms and signs resolved within 4 weeks, then erdafitinib was resumed at 2 dose levels lower than the current dose  This dose level was monitored for recurrence  If recurrence was observed, erdafitinib was permanently discontinued |
| **Grade 4**  Sight-threatening consequences; blindness (20/200 or worse). | Immediate and permanent erdafitinib discontinuation | Continued ophthalmologist monitoring until complete resolution or stabilization |

FGFRi-associated retinopathy, FGFR inhibitor-associated retinopathy.
