# Supplementary material for: Incidence and outcomes of FGFR inhibitor-associated retinopathy of patients treated with oral erdafitinib across the clinical trial program
Source: Oncologist. 2026 May 13;31(7):oyag174. doi: 10.1093/oncolo/oyag174 (PMC13293072; doi:10.1093/oncolo/oyag174)
Supplement: oyag174_Supplementary_Data [file oyag174_supplementary_data.zip › 22-May-2026_101752_OHagan_The_Oncologist_SupplementaryMaterial_17APR2026.docx]

Incidence and outcomes of FGFR inhibitor-associated retinopathy of patients treated with oral erdafitinib across the clinical trial program

Anne O'Hagan, Arlene Siefker-Radtke, Yohann Loriot, Kris Deprince, Lauren Crow, Michal Laron, Ron Adelman, Hussein Sweiti, Spyros Triantos

Supplementary Material

Supplementary Table S1. Management of FGFRi-associated retinopathy used across oral erdafitinib clinical studies

Severity and grading	Immediate action	Dose management
**Grade 1**<br>**Asymptomatic or mild symptoms; clinical or diagnostic observations only, or abnormal Amsler grid test**	• Refer to an ophthalmologist<br>• If an ophthalmologist evaluation was not performed within 7 days, erdafitinib was immediately withheld until an ophthalmologist evaluation was performed	• If diagnosis from ophthalmologist evaluation was retinal abnormality, erdafitinib was withheld until signs, symptoms, and ocular findings were resolved<br>• If signs and symptoms resolved within 4 weeks of ophthalmologist evaluation, erdafitinib was resumed at the next lower dose level<br>• If no recurrence was observed at this dose level after 1 month, then erdafitinib dose re-escalation was considered
**Grade 2**<br>**Moderate; limiting age-appropriate instrumental activities of daily living**	• Refer for an ophthalmologist evaluation<br>• Immediately withhold erdafitinib	• If there was no evidence of FGFRi-associated retinopathy, erdafitinib was continued at the next lower dose level upon resolution<br>• If signs, symptoms, and ocular findings resolved within 4 weeks of ophthalmologist evaluation, then erdafitinib was resumed at the next lower dose level<br>• This dose level was monitored for recurrence
**Grade 3**<br>**Severe or medically significant but not immediate sight threatening; limiting self-care activities of daily living**	• Refer for an ophthalmologist evaluation<br>• Immediately withhold erdafitinib	• If symptoms and signs resolved within 4 weeks, then erdafitinib was resumed at 2 dose levels lower than the current dose<br>• This dose level was monitored for recurrence

Severity and grading	Immediate action	Dose management
		• If recurrence was observed, erdafitinib was permanently discontinued
**Grade 4**<br>**Sight-threatening consequences; blindness (20/200 or worse)**	Immediate and permanent erdafitinib discontinuation	Continued ophthalmologist monitoring until complete resolution or stabilization
